# The effectiveness of thoracic medial branch radiofrequency neurotomy using a three-tined electrode: A single-arm, retrospective cohort study

**DOI:** 10.1016/j.inpm.2025.100563

**Published:** 2025-03-01

**Authors:** Hasan Sen, Amanda Cooper, Andrew Stephens, Brook Martin, Robert S. Burnham, Aaron Conger, Zachary L. McCormick, Taylor R. Burnham

**Affiliations:** a Department of Physical Medicine and Rehabilitation, University of Utah, Salt Lake City, UT, United States; b Department of Physical Medicine and Rehabilitation, University of Rochester Medical Center, Rochester, NY, United States; c Department of Orthopedics, University of Utah, Salt Lake City, UT, United States; d Division of Physical Medicine and Rehabilitation, University of Alberta, Edmonton, AB, Canada; e Vivo Cura Health, Calgary, AB, Canada; f Central Alberta Pain and Rehabilitation Institute, Lacombe, AB, Canada; g Division of Physical Medicine and Rehabilitation, University of Calgary, Calgary, AB, Canada

**Keywords:** Radiofrequency ablation, Thoracic medial branch, Facet joint pain

## Abstract

**Background:**

Thoracic medial branch radiofrequency neurotomy (TMBRFN) is used to treat chronic thoracic facet joint pain, but research on its technique and effectiveness is still needed. The current International Pain and Spine Intervention Society Practice Guidelines do not describe a technique for TMBRFN.

**Objectives:**

Evaluate the effectiveness of TMBRFN in patients with thoracic facet joint pain.

**Methods:**

Single-arm, retrospective cohort study of consecutive patients from two Canadian musculoskeletal pain management clinics who underwent first-time TMBRFN between 2016 and 2022. The primary outcome was the proportion of patients with ≥50 % reduction in numerical rating scale (NRS) pain score at 3-months post-procedure. Secondary outcomes included the proportion of patients who achieved a ≥17-point reduction on the Pain Disability Quality-Of-Life Questionnaire-Spine (PDQQ-S) at 3-months, as well as mean patient-reported percentage pain relief and duration of relief after a successful index TMBRFN in individuals who reported a return of their index symptoms.

**Results:**

18 consecutive patients (50.0 % male; mean age 60.9 ± 15.3 years; mean BMI 30.3 ± 6.9 kg/m^2^) were analyzed. At 3 months post-procedure, 10 patients (55.6 % [95%CI 33.7–75.4]) reported ≥50 % NRS pain reduction and 9 (50.0 % [95%CI 29.0–71.0]) reported ≥17-point PDQQ-S reduction. Of the 10 patients with successful treatment responses, 4 had a return of symptoms after an average of 9.3 ± 2.2 months with a reported retrospective mean percentage pain relief of 70.0 ± 34.6 %.

**Conclusion:**

Within this cohort, approximately 60 % of patients experienced improvement in pain and disability at 3 months following TMBRFN. Among patients whose index symptoms returned after successful treatment, the average reported pain relief was 70 % for close to 9 months. Larger, prospective studies with long-term outcomes are needed to better elucidate the safety and effectiveness of TMBRFN.

## 1 Introduction

Spinal pain is a widely acknowledged health issue. The occurrence of spinal pain at some point in a person's life has been reported to range from 54 % to 80 % [1]. The lumbar and cervical spine are the most studied spinal regions, likely due in part to their propensity for degenerative changes, which are often associated with pain, work-related injuries, headaches, psychosocial disturbances, and significant healthcare costs [2,3]. The healthcare burden of spine pain continues to grow, as demonstrated by Dieleman et al. [4,5], with spending increasing by an estimated 53.5 % from $87.6 billion in 2013 to $134.5 billion in 2016.

The lifetime prevalence of thoracic spine pain is estimated to be approximately 15–20 % [6,7]. Because of this, the thoracic spine has received less attention in terms of clinical research in comparison to the lumbar and cervical spine, yet pain experienced in the thoracic spine can be equally disabling [2]. Zygapophyseal or “facet” joints are implicated in about one-third to half of patients with non-radiating thoracic pain, similar to the cervical and lumbar regions [8].

Radiofrequency neurotomy (RFN) is an effective treatment for reducing axial spine pain, with most clinical research evaluating cervical [[9], [10], [11]] and lumbar [12,13] RFN. Thoracic medial branch radiofrequency neurotomy (TMBRFN) is utilized to treat chronic thoracic facet joint pain. TMBRFN uses radiofrequency energy to disrupt the function of a thoracic medial branch nerve, preventing it from sending pain signals from a symptomatic facet joint to the brain. Despite a notable gap in clinical research on thoracic medial branch radiofrequency neurotomy (TMBRFN), limited evidence suggests it may be used to effectively treat facet-mediated thoracic spine pain. Studies by Rohof and Stolker et al. have shown that more than 80 % of patients reported ≥50 % pain reduction following TMBRFN treatment [14,15]. Additionally, Speldewinde demonstrated that 68 % of patients had ≥50 % pain relief with this procedure [16]. However, these studies are limited by small sample sizes, lack of control groups and potential selection bias; therefore, further research is essential.

Furthermore, the current Practice Guidelines of the International Pain and Spine Intervention Society (IPSIS) do not outline a specific technique for TMBRFN, primarily due to the intricate and potentially variable innervation of thoracic facet joints, which may differ from the original neuroanatomical descriptions by Bogduk and Chua [7,[17], [18], [19], [20], [21], [22]]. This underscores the need for further investigation and standardization in this area.

This study aims to help fill the literature gap regarding chronic thoracic facet joint pain management by (1) evaluating patient-reported outcomes of TMBRFN performed with a three-tined electrode and perpendicular approach and (2) contributing to the limited evidence available to help guide clinical decision-making in this underexplored area.

## 2 Methods

### 2.1 Data collection

This retrospective cohort study was conducted at two Canadian musculoskeletal and pain clinics. The electronic medical records of consecutive patients who underwent TMBRFN between 2016 and 2022 were reviewed. Local approval by the Conjoint Health Research Ethics Board at the University of Calgary (Ethics ID#: REB20-0355) was obtained.

Data extraction was performed by the author (R.B.). The inclusion criteria were: (a) thoracic pain that had been refractory to conventional conservative treatment, (b) clinical features suggestive of facetogenic thoracic pain such as localized pain and tenderness upon palpation over the thoracic facet joints, (c) fluoroscopically guided medial branch blocks (MBB) ± intra-articular blocks (IAB) resulting in ≥50 % pain relief, (d) Numerical Rating Scale (NRS) pain and Pain Disability Quality-Of-Life Questionnaire-Spine (PDQQ-S) scores recorded at baseline and 3-months post-intervention, and (e) first-time TMBRFN procedure. For criterion (c), the response to prognostic blocks was determined by having patients complete a 0–10 scale NRS pain diary. Recordings were made just before and at 30-min intervals for 6 h post-block, and this diary was sent back to our clinics for review. The maximal pain relief during this period was calculated from the pre-versus post-prognostic block NRS pain scores. Patients were included in the study if they achieved ≥50 % pain relief at any time within these 6 h. Additionally, patients were asked if they experienced any functional improvement or enhanced ability to participate in specific activities, though this was not used as an inclusion criterion.

The exclusion criteria were: (a) diagnostic block(s) performed offsite (b) confounding interventions or injuries (*i.e.*, epidural or facet joint steroid injection), in the interim between TMBRFN and 3-month follow-up, and (c) TMBRFN procedures concurrently with another intervention on the same date.

### 2.2 TMBRFN procedures

#### 2.2.1 Patient positioning and anxiety/pain management

Patients undergoing TMBFRN were positioned prone on the fluoroscopy table and the area was prepped and draped in the usual sterile fashion. Heart rate and oxygen saturation were monitored throughout the procedure. Preprocedural sublingual lorazepam (1–2 mg) was provided for patients with anxiety. If required for additional analgesic, self-administered inhaled nitrous oxide was used.

#### 2.2.2 RFN electrode target sites

Final electrode placement was based on historical dissection data describing thoracic medial branch anatomy [17,18]. These sources describe a consistent relationship between the nerves and the superior-lateral corner of the transverse process of the T1–4 and T10–11 vertebra, respectively. Therefore, the target point used for the associated medial branch nerves was the dorsal surface of the transverse process, slightly medial to its superolateral corner. For T4–8 medial branches, no bony landmark can serve as a target point. These branches are displaced somewhat superiorly to the transverse process, suspended in the intertransverse space. Dissection studies have shown that the nerve can be found dorsal to the rib but at the depth of transverse process of the T5–9 vertebrae, respectively, cephalad of the target point for medial branches at other levels [17,18]. Therefore, the target point for these nerves is just above the superolateral corner border of the transverse process, at the depth of transverse process. For the T11–12 medial branches, the target site is similar to the lumbar region, located at the junction of the superior articular process and the transverse process of the T12 and L1 vertebrae, respectively.

#### 2.2.3 RFN cannula placement and lesioning

**Fig. 1:**
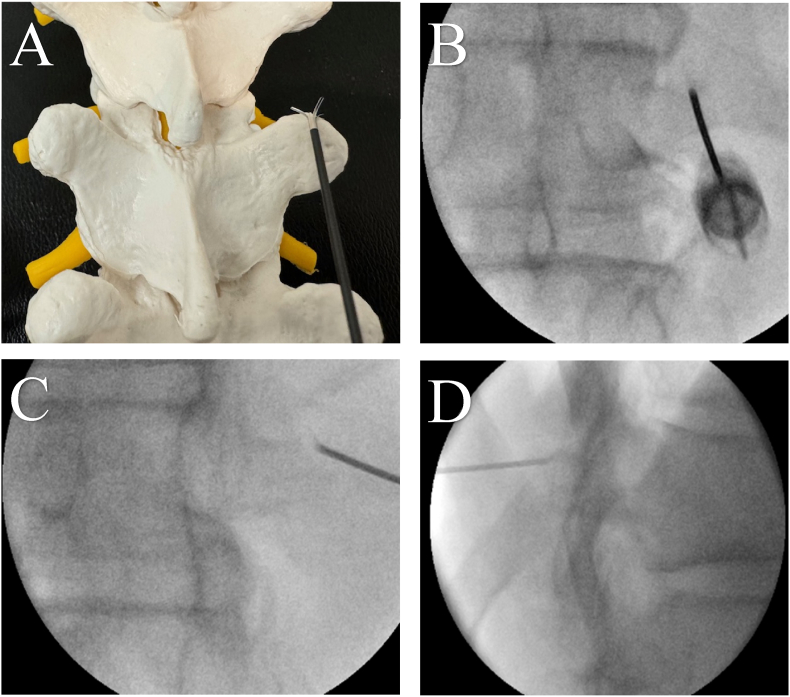
Images of TMBRFN procedure showing cannula placement for targeting right T10 medial branch nerve. (A) AP view of anatomical model. (B) AP view on fluoroscopy. (C) CLO view. (D) Lateral view. AP = Anteroposterior; CLO = Contralateral Oblique; TMBRFN = Thoracic Medial Branch Radiofrequency Neurotomy.

AP, contralateral oblique and lateral fluoroscopic views were used for C8–T10 medial branch nerve lesioning (Fig. 1). After the target was identified, the skin was anesthetized just below the target site by raising a skin wheal with 0.5 ml of 1 % lidocaine, using a 30-gauge needle. Under fluoroscopic guidance, the area of the medial branch nerve and the overlying soft tissue were anesthetized using a 25-gauge needle. Next, an 18-gauge 100 mm Trident (Diros) multi-tined cannula with a 5 mm active tip was advanced to the target under fluoroscopic guidance. The superior edge of the transverse process was contacted with the bevel facing inferiorly. The cannula was then advanced 1–2 mm, sliding posteriorly along the superior edge of the transverse process. The bevel was then rotated superiorly, and the tines were deployed. With the Trident cannula in this position, it was anticipated that the exposed cannula tip and proximal portions of the deployed tines would capture the C8–T3 and T9–T10 medial branch nerves whereas the distal portion of the deployed tines would capture the more cephalad located T4–T8 medial branch nerves. Finally, 1.0 ml of 1 % lidocaine was injected at the target sites and radiofrequency was initiated at 80 °C for 90 s after a 15-s ramp up time.

For the T11 and T12 medial branches, the procedure started with an AP view to identify the T12 and L1 vertebrae, respectively. An oblique view was subsequently obtained in order to identify a “scotty dog” view in order to access the junction between the superior articular process and the transverse process. After identifying the target, the skin was anesthetized by raising a skin wheal with 0.5 ml of 1 % lidocaine, using a 30-gauge needle. Through a 25-gauge needle and under fluoroscopic guidance, the area of the medial branch nerve and the overlying soft tissue were anesthetized. Next, an 18-gauge 100 mm Trident (Diros) multi-tined cannula with a 5 mm active tip was advanced to the target. Optimal needle placement was confirmed with AP and lateral views and then the tines were deployed. Finally, 1.0 ml of 1 % lidocaine was injected at the target sites and radiofrequency was initiated at 80 °C for 90 s after a 15-s ramp up time. All patients tolerated the procedure.

#### 2.2.4 Post-procedure care

Patients were monitored for post-procedure complications for 30 min in the recovery area. Prior to discharge, each patient underwent lung examination and also received instructions regarding symptoms of pneumothorax and the need for urgent evaluation, including chest x-ray, should they develop.

### 2.3 Outcome assessment

Patient selection paradigms were categorized based on the number (single vs. dual) and type (MBB vs. IAB) of prognostic blocks, as well as subsequent percentage pain relief based on patient-reported NRS pain scores collected pre-versus post-block. Percentage pain reduction from baseline in response to blocks was calculated using the lowest NRS pain score recorded within 6 h post-injection. Using this schema, six block criteria were established: MBB/MBB ≥80 %, MBB/MBB 50–79 %, IAB/MBB ≥80 %, IAB/MBB 50–79 %, MBB ≥80 %, and MBB 50–79 %.

Outcomes for pain and function were evaluated at 3 months post-procedure using an 11-point NRS scale and the Pain Disability and Quality of Life Questionnaire-Spine (PDQQ-S), respectively. The PDQQ-S is a validated six-question patient-reported outcome measure designed for use in the field of minimally invasive interventional spine care, for which the minimal clinically important difference (MCID) is a score decrease of ≥17 points from baseline [23,24]. The primary study outcome used to determine treatment success was the proportion of patients with ≥50 % NRS pain score reduction at 3 months. Secondary outcomes included the proportion of patients reporting a ≥17-point PDQQ-S reduction at 3 months, as well as mean percentage reduction in NRS pain scores, and mean duration of pain relief in months among patients who returned for repeat TMBRFN upon recurrence of their index symptoms after a successful TMBRFN procedure.

### 2.4 Data analysis

Data analysis included descriptive statistics, with calculation of means/standard deviations for continuous variables and frequencies/percentages for categorical variables. A 95 % confidence interval was also calculated for the proportions of participants with ≥50 % NRS reduction and ≥17-point PDQQ-S reduction.

## 3 Results

18 consecutive patients (50.0 % male; mean age 60.9 ± 15.3 years; mean BMI 30.3 ± 6.9 kg/m^2^) met the inclusion criteria and were analyzed. Participant demographic, clinical, and procedural variables are presented in Table 1. Distribution of the six block criteria was as follows: (1) 33.3 % MBB/MBB ≥80 %, (2) 5.6 % MBB/MBB 50–79 %, (3) 27.8 % IAB/MBB ≥80 %, (4) 5.6 % IAB/MBB 50–79 %, (5) 5.6 % MBB ≥80 %, and (6) 22.2 % MBB 50–79 %.

**Table 1:** Participant demographics, clinical characteristics, and procedural variables (*N* = 18).

Variable	No. (%)
**Gender**	
Male	9 (50.0)
Female	9 (50.0)
**Smoking**	
Yes	2 (13.3)
No	13 (86.7)
*Unknown*	*3*
**Exercise**	
Yes	6 (42.9)
No	8 (57.1)
*Unknown*	*4*
**Working**	
Yes	7 (53.9)
No	3 (23.1)
Retired	3 (23.1)
*Unknown*	*5*
**Workup**	
Internal	14 (77.8)
External	4 (22.2)
**Clinic**	
Practice 1	4 (22.2)
Practice 2	14 (77.8)
**Diagnostic block paradigm**^a^	
MBB/MBB ≥80 %	6 (33.3)
MBB/MBB 50–79 %	1 (5.6)
IAB/MBB ≥80 %	5 (27.8)
IAB/MBB 50–79 %	1 (5.6)
MBB ≥80 %	1 (5.6)
MBB 50–79 %	4 (22.2)
**Laterality**	
Unilateral	10 (55.6)
Bilateral	8 (44.4)
**Total number of levels targeted**	
1	1 (5.6)
2	6 (33.3)
3	3 (16.7)
4	2 (11.1)
5	1 (5.6)
6	3 (16.7)
7	1 (5.6)
8	1 (5.6)

**Age in yr** (*n* = 18); mean (SD)	60.9 (15.3)
**Body mass index in kg/m**^**2**^ (*n* = 14); mean (SD)	30.3 (6.9)
**Pain duration in yr** (*n* = 15); mean (SD)	12.6 (12.7)
**NRS at baseline** (*n* = 18); mean (SD)	7.7 (1.2)
**PDQQ-S at baseline** (*n* = 18); mean (SD)	49.2 (5.5)

IAB = Intra-articular Block; MBB = Medial Branch Block; NRS = Numeric Rating Scale; PDQQ-S = Pain Disability and Quality of Life Questionnaire-Spine; SD = Standard Deviation.

a Diagnostic block paradigms are categorized according to block number (singe vs. dual), block type (IAB vs. MBB), and subsequent patient-reported percentage pain relief.

**Fig. 2:**
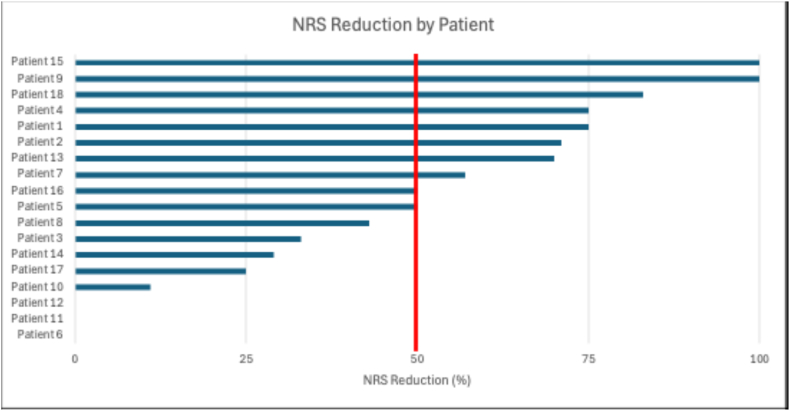
Tornado diagram of percentage NRS pain score reduction at 3-month follow-up after TMBRFN. Each horizontal bar represents an individual patient. NRS = Numeric Rating Scale; TMBRFN = Thoracic Medial Branch Radiofrequency Neurotomy.

**Fig. 3:**
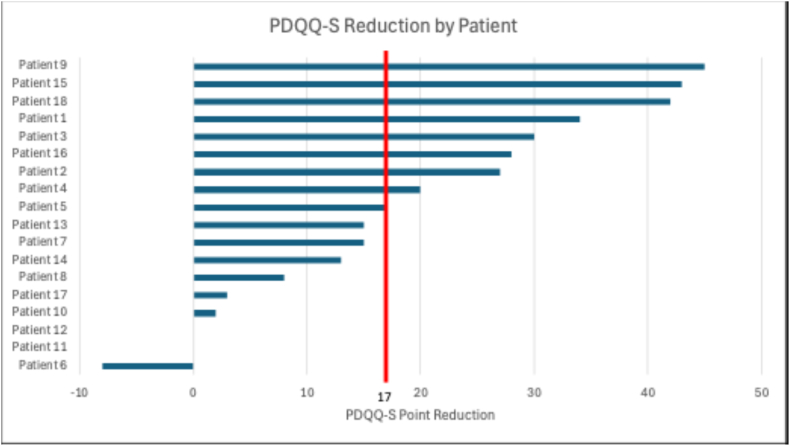
Tornado diagram of PDQQ-S score reduction at 3-month follow-up after TMBRFN. Each horizontal bar represents an individual patient. PDQQ-S = Pain Disability and Quality of Life Questionnaire-Spine; TMBRFN = Thoracic Medial Branch Radiofrequency Neurotomy.

At 3 months post-procedure, 10 patients (55.6 % [95%CI 33.7–75.4]) reported ≥50 % NRS pain reduction and 9 (50.0 % [95%CI 29.0–71.0]) reported ≥17-point PDQQ-S reduction (Fig. 2, Fig. 3; Table 2). Of the 10 patients with successful treatment responses, 4 had a return of symptoms after an average of 9.3 ± 2.2 months with a reported retrospective mean percentage pain relief of 70.0 ± 34.6 %. No adverse events were reported.

**Table 2:** Primary and secondary outcomes for pain reduction, functional improvement, and duration of relief.

Outcome Variable	No. (%)	95 % CI (yes)
**≥ 50 % NRS reduction** (*n* = 18)		
Yes	10 (55.6)	33.7, 75.4
No	8 (44.4)	
**≥ 17 PDQQ reduction** (*n* = 18)		
Yes	9 (50.0)	29.0, 71.0
No	9 (50.0)	

**NRS at 3 months** (*n* = 18); mean (SD)	4.0 (2.6)	
**PDQQ-S at 3 months** (*n* = 18); mean (SD)	30.6 (17.2)	

**Retrospective percentage pain relief** (*n* = 4); mean (SD)	70.0 (34.6)	
**Retrospective duration of improvement in months** (*n* = 4); mean (SD)	9.3 (2.2)	

CI = Confidence Interval; NRS = Numeric Rating Scale; PDQQ-S = Pain Disability and Quality of Life Questionnaire-Spine; SD = Standard Deviation.

## 4 Discussion

Our findings demonstrate that most (∼60 %) of those who underwent first-time TMBRFN achieved clinically significant pain relief and functional improvement at 3 months. Approximately 60 % of participants reported ≥50 % reductions in NRS pain scores, and 50 % reported ≥17-point reductions in PDQQ-S scores at three months. The average duration of therapeutic benefit was approximately 9 months among treatment responders whose index symptoms returned. The use of a multi-tined cannula to perform TMBRFN is innovative, and to our knowledge, our study is the first to report on the use of three-tined electrode in TMBRFN. Our results add to the limited literature supporting TMBRFN as an effective treatment for thoracic facet pain.

Among the few published studies on TMBRFN that are available for comparison, our results are most similar to those of Speldewinde, who demonstrated that 68 % of 28 monopolar TMBRFN procedures resulted in a ≥50 % pain reduction lasting longer than 2 months [16]. These findings are less robust than other literature reports of TMBRFN outcomes. In a retrospective analysis of 71 patients treated with bipolar RFN, Rohof et al. found that over 80 % experienced ≥50 % NRS pain score reduction at both 3- and 12-months post-procedure [14]. Similarly, Stolker et al. reported a case series involving 40 patients who underwent conventional RFN, with approximately 80 % of participants reporting ≥50 % pain relief at 2-month follow-up and at an average long-term follow-up of 31 months [15].

There are several potential explanations for these discrepancies. One key factor could be differences in the technical approach to TMBRFN. In our study, either three-tined or conventional monopolar electrodes were utilized to create a single lesion per target. The study by Rohof et al. employed a bipolar RF technique, which may have resulted in comparably larger lesion sizes. Given the complex and potentially variable innervation of the thoracic facet joints, a larger lesion size might be necessary to ensure more comprehensive nerve ablation, as thoracic facet joint neuroanatomy is more intricate than initially described in earlier works like those by Chua and Bogduk [17,18]. Recent research by Talsma et al. further highlights the more nuanced anatomical variations in thoracic zygapophysial joint innervation, suggesting that a better understanding of this complex neuroanatomy may help optimize treatment techniques and improve outcomes [20].

Thoracic pain is relatively uncommon compared to lumbar and cervical pain, as described in the literature [6]. The complexity of thoracic anatomy contributes to these discrepancies [17,18,21]. The intricate structure of the thoracic region includes the rib cage, numerous muscles, and critical organs, making diagnosing and treating thoracic pain more challenging [25]. Despite advances in medical research and technology, our understanding of thoracic anatomy continues to evolve. Talsma et al. challenged the traditional understanding of thoracic medial branch neuroanatomy, as described by Chua and Bogduk [17,18], through a single cadaveric dissection [20]. Their findings, supported by Ishizuka and Joshi et al. [21,22], highlighted variability in the location and course of thoracic medial branches, particularly at T4–8, where no clear bony landmarks can reliably serve as target points. These branches are positioned superior to the transverse process, within the intertransverse space, and typically found dorsal to the rib at the depth of the transverse processes. As our understanding of thoracic facet neuroanatomy improves, the targets for TMBRFN will also be refined, potentially leading to better outcomes.

### 4.1 Limitations

This study has several limitations that must be acknowledged. One limitation is the short follow-up period of only 3 months post-intervention, restricting our ability to adequately evaluate the long-term effects or durability of TMBRFN. Additionally, we had a small sample size of 19 patients, which may constrain generalizability of our findings. Lastly, as a single-arm retrospective cohort study, the lack of a comparison or control group limits our ability to definitively substantiate the effect of TMBRFN and adequately control for confounding variables. Given the relatively limited data in the literature regarding TMBFRN, our results provide additional insight into the potential of TMBRFN to alleviate pain and improve function in those with chronic thoracic facet joint pain.

## 5 Conclusion

Within this cohort, we observed reductions in pain and disability in approximately 60 % of patients who underwent first-time TMBRFN at 3-month follow-up. Among treatment responders whose index symptoms returned, average reported pain relief was 70 % for close to 9 months. Larger, prospective studies with long-term outcomes are needed to better elucidate the safety and effectiveness of TMBRFN, as well as to optimize patient selection and procedural technique.

## Funding

None.

## Declaration of competing interest

The authors declare the following financial interests/personal relationships which may be considered as potential competing interests: Zachary L. McCormick reports a relationship with International Pain & Spine Intervention Society that includes: board membership. Zachary L. McCormick reports a relationship with Avanos Medical Inc that includes: consulting or advisory and funding grants. Zachary L. McCormick reports a relationship with Boston Scientific Corporation that includes: funding grants. Zachary L. McCormick reports a relationship with Relievant Medsystems Inc that includes: funding grants. Zachary L. McCormick reports a relationship with Saol Therapeutics that includes: consulting or advisory and funding grants. Zachary L. McCormick reports a relationship with Spine Biopharma that includes: funding grants. Zachary L. McCormick reports a relationship with SPR Therapeutics Inc that includes: funding grants. Zachary L. McCormick reports a relationship with Stratus Medical that includes: funding grants. Zachary L. McCormick reports a relationship with Stryker that includes: consulting or advisory. Zachary L. McCormick reports a relationship with OrthoSon that includes: consulting or advisory. Taylor Burnham reports a relationship with Diros Technology Inc that includes: funding grants. Aaron Conger reports a relationship with Stratus LLC that includes: funding grants. Robert Burnham reports a relationship with International Pain & Spine Intervention Society that includes: funding grants. Brook Martin, PhD is the founder and CEO of STATIX, LLC an independent research consulting company, and is a Deputy Editor of Evidence and Methods for The Spine Journal If there are other authors, they declare that they have no known competing financial interests or personal relationships that could have appeared to influence the work reported in this paper.
